# Evaluating Adherence to 2023 National Institute for Health and Care Excellence (NICE) Guidelines for Computed Tomography (CT) Scans in Head Injury Presentations at University Hospital of North Durham in England, UK: A Quality Improvement Project

**DOI:** 10.7759/cureus.96412

**Published:** 2025-11-09

**Authors:** Tahir Mushtaq, Dahiru Garkuwa

**Affiliations:** 1 Emergency Department, University Hospital of North Durham, Durham, GBR

**Keywords:** anticoagulant drugs, ct imaging in traumatic brain injury, emergency medicine, mild head injury, neuroradiology, nice guidelines, quality improvement projects, technology-enhanced education

## Abstract

Introduction

Traumatic brain injury (TBI) remains a major public health concern in the United Kingdom, with computed tomography (CT) head imaging essential for the early detection of intracranial injury. This study aimed to evaluate local compliance with the National Institute for Health and Care Excellence (NICE) criteria at University Hospital of North Durham and assess whether education-based intervention could improve adherence to national standards.

Methods

This quality improvement study was conducted in two cycles during the year 2023-2024 in our emergency department. Adult patients (aged ≥16 years) undergoing CT head scans for traumatic head injury were retrospectively reviewed. In the first cycle, baseline adherence to NICE criteria was assessed. After the data was analysed, targeted interventions were introduced, including structured teaching for doctors and nurses, visible posting of CT criteria in key clinical areas, as well as increased senior clinician oversight in triage and handover points. The second cycle evaluated post-intervention compliance using the same methodology. Data included clinical indications, documented risk factors, and timing of imaging. Statistical analysis compared the performance between the two cycles.

Results

Overall compliance with NICE guidelines improved from 29 (78.4%) of 37 patients in the first cycle to 46 (88.5%) of 52 in the second cycle. For high-risk patients, CT completion within one hour increased from 4/10 (40%) to 11/18 (61.1%). For patients on anticoagulation, compliance remained consistently high at 100% in both cycles. For patients with amnesia or loss of consciousness, imaging within eight hours improved from 6/9 (66.7%) to 22/28 (78.6%). These improvements were largely attributed to increased awareness, education, and better early identification of high-risk cases following intervention.

Conclusion

This study demonstrated that targeted interventions, such as staff education, visible guidelines, and senior oversight, improved compliance with NICE CT head guidelines for patients with head injury. Sustained changes in clinical practice can enhance patient safety and support more efficient emergency care.

## Introduction

Traumatic brain injury (TBI) remains a significant public health challenge in the United Kingdom, particularly among young adults, where it represents a leading cause of death and disability [1]. Each year, more than one million individuals in England and Wales attend emergency departments following a recent head injury, with around 200,000 requiring hospital admission and an estimated 40,000 diagnosed with an actual brain injury [2].

Computed tomography (CT) remains the mainstay imaging modality for evaluating patients with suspected TBI due to its rapid imaging capabilities and sensitivity in detecting skull fractures and acute intracranial haemorrhage [3]. The National Institute for Health and Care Excellence (NICE) initially published its guidance in 2004 by recommending CT scans as a superior imaging modality to skull radiographs. These were revised substantially in 2014 and again in 2023, offering more comprehensive and time-sensitive imaging criteria.

This quality improvement project (QIP) was conducted at University Hospital of North Durham in England, UK, between 2023 and 2024, with the aim of evaluating local adherence to the NICE 2023 guidelines for CT head imaging in patients presenting with head injuries. It also aimed to determine whether the introduction of targeted interventions, such as clinical education and improved access to guidelines, could enhance compliance with national standards and promote safer, evidence-based practice.

## Materials and methods

In this QIP, CT scans performed in patients aged 16 and over who presented with TBIs were evaluated and assessed for compliance in accordance with NICE guidelines. This project was registered and approved through the Clinical Audit Team of County Durham and Darlington NHS Foundation Trust (approval number: 1862) to ensure adherence with institutional standards. 

National guidance recommends that patients with head injury should receive urgent CT imaging if certain high-risk features are present. Scanning within one hour is required for patients with a Glasgow Coma Scale (GCS) score of 12 or less on arrival or if the score remains below 15 two hours after injury [4]. The same timeframe applies to patients with suspected open or depressed skull fracture, clinical signs of basal skull fracture (including haemotympanum, periorbital bruising, cerebrospinal fluid leak from the ear or nose, or Battle's sign), post-traumatic seizures, focal neurological deficits, or repeated vomiting.

For patients at lower but still important risk, CT imaging should be performed within eight hours. This includes patients aged 65 years or older with a history of loss of consciousness or amnesia, those with a clotting disorder or taking anticoagulant/antiplatelet treatment (excluding aspirin monotherapy) and who have had loss of consciousness or amnesia, as well as those injured by a dangerous mechanism (such as a pedestrian or cyclist struck by a vehicle, ejection from a vehicle, or a fall from more than one metre or five stairs). Imaging within eight hours is also indicated when retrograde amnesia of more than 30 minutes is present.

Patients on anticoagulant or antiplatelet therapy without any other indications should still be considered for CT head imaging, ideally within eight hours of injury, or within one hour if they present more than eight hours after the event.

The QIP standard was set at 100%: all CT head scan requests for eligible patients should have documented head injury risk factors in accordance with NICE guidelines.

First cycle

The first audit cycle involved retrospective data collection over a one-month period in 2023. Inclusion criteria were all adult patients (aged ≥16 years) presenting with head injury who underwent CT head scanning during the audit period. Exclusion criteria included scans performed for non-traumatic indications such as stroke, seizure, or metabolic encephalopathy. A total of 97 head injury cases were reviewed; 37 patients met the inclusion criteria and underwent CT scans. Data were collected from electronic patient records (ePR) and imaging requests and entered into Microsoft Excel (Microsoft Corporation, Redmond, Washington, United States). Clinical indications, documented risk factors, and timing of imaging were analysed against the guideline standards.

Intervention

**Figure 1:**
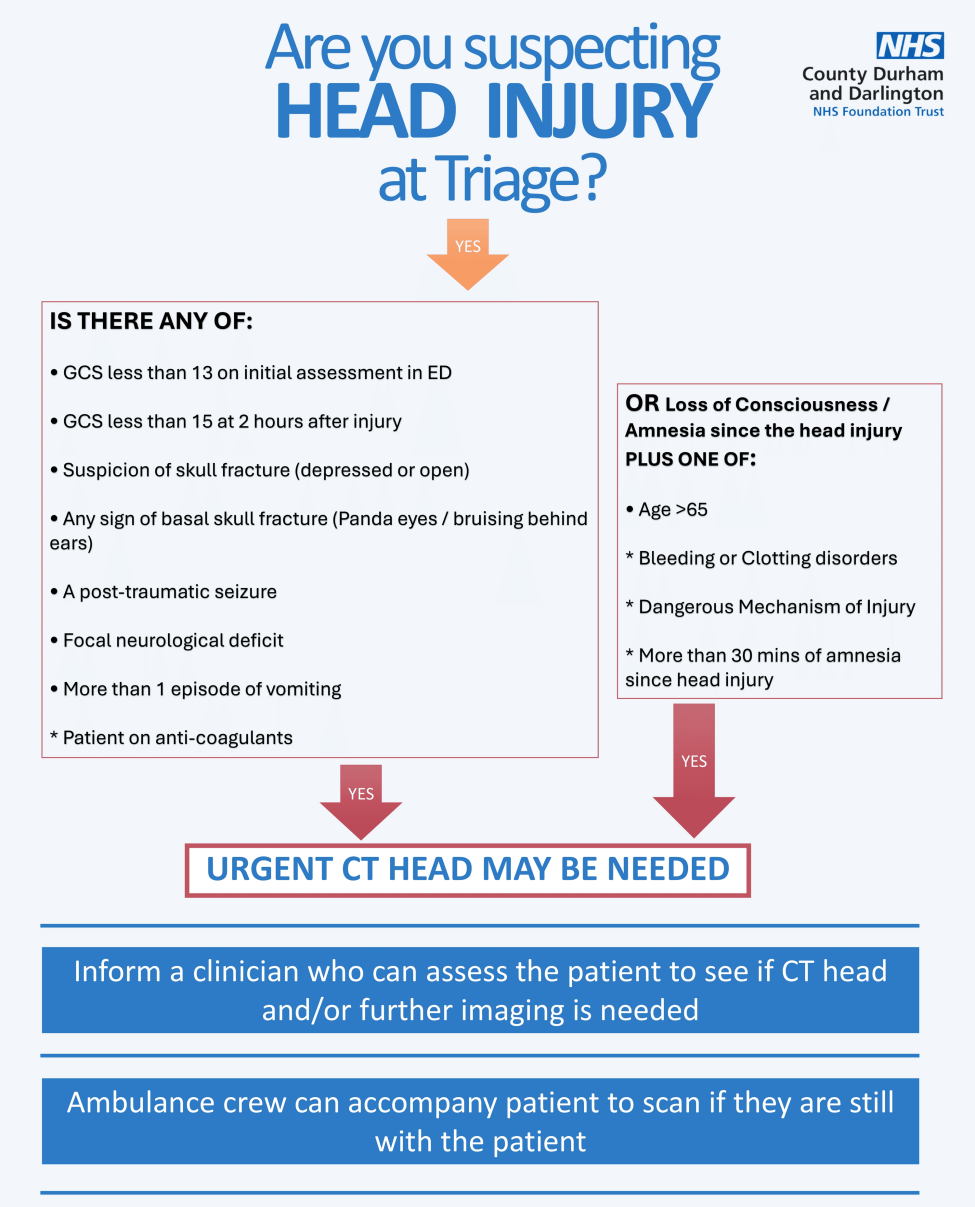
Head injury poster GCS: Glasgow Coma Scale; ED: emergency department; CT: computed tomography Image Credit: Tahir Mushtaq

Following the first cycle, a series of targeted interventions were introduced to improve guideline compliance. The audit findings had highlighted that the earliest point of contact, specifically triage and the ambulance handover area, plays a critical role in recognising risk factors for CT imaging. Therefore, it was important to educate staff working in these areas. At our district general hospital, CT scans for traumatic head injuries are protocol-driven and do not require formal approval from the radiology department; however, senior emergency medicine input is still required. The emergency department is predominantly staffed by foundation year and senior house officer (SHO) doctors, many of whom have limited prior experience in emergency medicine. This underscored the need for focused educational sessions to improve awareness of the relevant guidelines. Additionally, the high turnover of patients in areas like the ambulance handover area prompted recognition of the need for senior clinical presence in such areas of the department. Following the presentation of the first audit cycle at a clinical governance meeting, and with the support of the department's educational consultant, structured teaching sessions were delivered to doctors. These encouraged regular reference to the CT head injury guidelines available on the hospital's clinical pathways and guidelines portal. In parallel, educational sessions were also arranged for nursing staff, in collaboration with the nurse in charge, to improve the early recognition of high-risk cases during triage and ambulance handover. To strengthen early risk assessment, a rapid assessment and treatment senior (RATS) clinician was assigned to oversee injury presentations, facilitating the timely identification of patients requiring urgent scans. Furthermore, visible summaries of NICE guidelines were developed and placed prominently in the triage area, ambulance handover area, and staff base to act as a quick reference for frontline staff (Figure 1).

Second cycle

The second audit cycle was conducted over a two-week period in early 2024, after the interventions had been implemented. The same data collection methods and inclusion criteria were used. In this cycle, 52 patients with head injuries were reviewed, all of whom underwent CT head scans. Of these, 46 cases met criteria for assessment under the NICE guidelines. As in the first cycle, data was analysed for documentation of indications, recognition of risk factors, and scan timing. Importantly, the audit design and sample sizes in both cycles were informed by the Royal College of Radiologists (RCR) AuditLive template titled "Compliance with NICE Guidelines 2014 for Traumatic Head Injury in Regard to CT" and exceeded the minimum standards recommended for local audits evaluating CT head scan compliance in trauma patients [5].

## Results

During the first cycle of the audit, conducted over a one-month period in 2023, 97 patients presenting with head injuries were reviewed. Of these, 37 (38.1%) patients underwent CT head scans that were evaluated against the NICE criteria. The second cycle, conducted over a two-week period in 2024 following an intervention, included 52 head injury patients, all of whom underwent CT imaging. Of these, 46 (88.5%) met the inclusion criteria for assessment under the audit standards.

In the first cycle, guideline adherence was suboptimal. Among the 37 patients, 29 (78.4%) were assessed using NICE guidelines, and eight did not receive CT imaging within the timeframe recommended by the guidelines, resulting in an overall compliance rate of 78.4%. In contrast, the second cycle demonstrated an improvement in practice, with only six (11.5%) of the 46 eligible patients receiving delayed imaging, raising overall compliance to 88.5% (Table 1).

**Table 1:** Comparison of adherence to guidelines "-" indicates not applicable. The chi-squared test was performed on the comparison between the two cycles.

Audit cycle	Adherent (n/%)	Non-adherent (n/%)	Total	Compliance (%)	Chi-square	P-value
First cycle	29 (78.4%)	8 (21.6%)	37	78.4%	-	-
Second cycle	46 (88.5%)	6 (11.5%)	52	88.5%	0.98	0.32

In the first cycle, 10/37 (27%) patients met the one-hour CT criteria. Of these, 5/10 (50%) had CT requested, and 4/10 (40%) had it completed within the recommended timeframe. In the second cycle, 18/52 (34.6%) patients met the one-hour CT scan criteria. Of these, 12 patients (66.7%) had a CT requested within one hour of risk recognition. Eleven patients (61.1%) had a CT scan completed within the one-hour window (Table 2).

**Table 2:** Comparison of compliance in patients requiring CT head scan within one hour "-" indicates not applicable. The chi-squared test was performed on the comparison between the two cycles. CT: computed tomography

Audit cycle	Required in 1 hour (n)	Requested in time (n/%)	CTs performed in time (n/%)	Performance (%)	Chi-square	P-value
First cycle	10	5/10 (50%)	4/10 (40%)	40%	-	-
Second cycle	18	12/18 (66.7%)	11/18 (61.1%)	61.1%	0.46	0.50

Compliance with the eight-hour cutoff as per the guideline was also assessed. In the first cycle, all 19 patients on anticoagulant therapy received timely CT scans, achieving 100% compliance. Among nine patients presenting with amnesia or loss of consciousness, six (66.7%) received imaging within eight hours. In the second cycle, 22 anticoagulated patients again achieved 100% compliance, while 22/28 (78.6%) patients with amnesia or loss of consciousness received CT scans within the recommended timeframe (Table 3).

**Table 3:** Comparison of eight-hour CT head scan compliance in specific patient subgroups "-" indicates that the test is not applicable due to identical outcomes in both cycles. CT: computed tomography; LOC: loss of consciousness

Patient subgroup	First cycle (n/%)	Second cycle (n/%)	Chi-square	P-value
Anticoagulated	19/19 (100%)	22/22 (100%)	-	-
Amnesia/LOC	6/9 (66.7%)	22/28 (78.6%)	0.08	0.78

These findings reflect overall improvement in clinical practice following the intervention, with notable increases in compliance for urgent one-hour scan requests, sustained high compliance for anticoagulated patients, and an overall rise in adherence to national imaging guidelines for TBI.

## Discussion

The initial audit cycle revealed room for improvement, particularly in recognising and acting upon high-risk features that required urgent scanning. Following the implementation of a series of targeted educational and structural interventions [6], the second audit cycle demonstrated an improvement in overall adherence to national standards; this aligns with evidence that shorter times to CT can benefit patient outcomes [7]. Although the differences between cycles did not reach statistical significance, the trends suggest better recognition of high-risk cases and more timely imaging.One of the most encouraging outcomes was the increased compliance with the one-hour CT scan target, indicating improved recognition and prompt action for time-sensitive cases. Importantly, although still below the ideal standard of 100%, this represents meaningful progress in a challenging clinical environment where timely decision-making is often impacted by system pressures [8] and staffing limitations.

Sustained 100% compliance was observed across both audit cycles for anticoagulated patients requiring CT within eight hours, a reassuring finding that suggested strong clinical awareness of the risks associated with head trauma in this subgroup [9]. Notably, none of these patients exhibited significant neurological findings; their GCS scores were either unchanged or consistent with baseline. The extended eight-hour window provided sufficient time to carry out thorough clinical assessments and arrange imaging without compromising care.

Meanwhile, moderate improvement was noted in patients presenting with amnesia or loss of consciousness; although not a statistically significant increase, it suggested a positive trend following educational reinforcement. The multifaceted intervention strategy appears to have had a beneficial impact. Structured teaching for resident doctors, who make up the bulk of our department's workforce, seems to have improved familiarity with the guidelines. Likewise, the emphasis on early recognition of high-risk features during ambulance handover and triage, supported by nursing education and the introduction of visible guideline summaries, likely contributed to earlier escalation and scan requests [10]. The involvement of a RATS clinician in the initial assessment of injury cases also helped bridge gaps in early senior decision-making.

When compared with findings from similar audits conducted in major trauma and district general hospitals, the results of the first cycle are consistent with observed patterns of poor adherence with NICE guidance and the need for further education and awareness of head injury clinical guidelines [11,12]. Previous studies have shown that targeted teaching and simple visual aids, such as printed guideline summaries, can improve scan timeliness and overall compliance with standards. For example, a 2020 quality improvement project at Wexford General Hospital demonstrated a rise in one-hour CT compliance, similar to the improvement observed in our audit from 4 (40%) to 11 (61.1%) patients meeting one-hour scan targets following the implementation of structured triage tools and educational sessions, despite only modest improvements in overall adherence [13]. While complete compliance remains difficult to achieve across many centres, our findings align with the broader evidence that structured, low-cost changes can produce measurable improvements in clinical practice [14].

Several limitations should be acknowledged. The retrospective nature of data collection means that some risk factors may have been present but not documented, potentially leading to the underestimation of compliance. The relatively small sample sizes, although above the RCR's recommended minimum for local audits, also limit the generalisability of the findings. Nonetheless, the audit offers a useful snapshot of current practice and the impact of pragmatic, achievable interventions.

It is also important to acknowledge the broader implications of over-scanning. Although CT head imaging is a critical diagnostic tool, unnecessary scans increase radiation exposure and place additional strain on healthcare resources [15]. In older adults, excessive imaging may contribute to prolonged emergency department stays, resulting in an increased risk of delirium [16]. This audit reinforces the value of clear guidelines in balancing the need for timely imaging with the risks of overuse.

Looking ahead, continued efforts are needed to embed these improvements into routine clinical practice. Regular refresher training of stakeholders involved, periodic re-auditing, and perhaps the introduction of prompts or checklists within the electronic patient record could further enhance compliance [17]. More broadly, investment in staffing and resources remains vital to support timely imaging in the increasingly pressured environment of emergency care.

## Conclusions

This QIP highlights a meaningful improvement in adherence to NICE guidelines for CT head imaging in TBI, particularly for patients requiring urgent scans. It also reinforces the value of targeted education, clinical leadership at the front door in the form of rapid assessment clinicians, and the use of visible guideline aids in improving patient safety and care quality.

These findings demonstrate that simple, focused interventions can lead to valuable changes in clinical practice. By embedding guideline awareness into everyday workflows, teams can act more promptly and confidently in high-risk situations. This project also underscores the importance of ongoing audit cycles to sustain improvements and adapt to evolving challenges. Our experience suggests that quality improvement in emergency care is both achievable and impactful, even in busy clinical settings.
